# An online, two-day educational seminar had no impact on disease-specific knowledge in patients with systemic sclerosis

**DOI:** 10.1038/s41598-024-64532-4

**Published:** 2024-06-14

**Authors:** Nancy Garbe, Katja Raberger, Andreas Wienke, Gernot Keyßer, Christoph Schäfer

**Affiliations:** 1 Department of Internal Medicine II, Rheumatology, University Hospital Halle (Saale), Ernst-Grube-Straße 40, 06120 Halle (Saale), Germany; 2 Clinic for Pediatrics I, University Hospital Halle (Saale), Halle (Saale), Germany; 3 Institute of Medical Epidemiology, Biometrics, and Informatics, Medical Faculty of the Martin Luther University Halle-Wittenberg, Halle (Saale), Germany

**Keywords:** Systemic sclerosis, Patient education, Self-help groups, Health knowledge, Systemic sclerosis, Health services

## Abstract

Systemic sclerosis (SSc) is a multifaceted disease, and its diagnosis triggers substantial anxiety and uncertainty for those affected. Currently, there are no valid data describing the impact of disease-specific patient education on the disease knowledge available. We created a two-day, online educational seminar to provide SSc patients with disease-specific information. The primary objective of the study was to observe the change in the disease-specific knowledge of the patients. A total of 118 patients were randomized into an intervention group and a waiting list control group. The change in knowledge was assessed using a multiple-choice test. The intervention group completed the questionnaire before, directly after, and 3 months after the seminar, while the waiting list control group also took the test 3 months before the seminar to rule out nonspecific learning. The primary outcome measure was the score difference between baseline and 3 months after baseline. The study was registered in the German Clinical Trials Register (protocol code DRKS00024915). The educational seminar resulted in a small, but measurable, increase in knowledge. While the two tests in the waiting list control group prior to the seminar did not show a nonspecific increase in disease knowledge, the intervention led to a numerical increase in knowledge (mean ± sd score difference 0.34 ± 1.31, 95% CI (− 0.23; 0.86), p = 0.26) that did not reach statistical significance. Multiple linear regression analysis showed that being a member of a self-help group (β = 1.12; p = 0.03) is a positive predictor of a higher disease knowledge. Although highly appreciated by participants, a two-day online seminar may not be the most appropriate format to generate measurable disease-specific knowledge. Self-help group membership was a positive predictor of a higher level of disease-specific knowledge prior to the educational seminar and should be recommended to every affected person.

## Introduction

The majority of inflammatory rheumatic diseases affect patients throughout their lifetime. Many entities, including systemic sclerosis (SSc), have a considerable impact on life expectancy and quality of life^1,2^. In addition to drug therapy, self-management by affected individuals has recently received increasing attention. Patients are required to manage physical and emotional challenges such as pain, fatigue, deformities, changes in countenance, and self-image^3^. For this purpose, patients need to have some basic knowledge about their complex disease, potential complications, and treatments^4,5^. In general, patient education has been shown to positively affect quality of life and self-management in patients with chronic conditions. It is therefore an integral part of rheumatic disease management according to the European League Against Rheumatism (EULAR) recommendations^6^. The health care system often offers professionally organized patient education programs for common diseases^7,8^, but offers far fewer for rare conditions^9,10^. A recent European survey by the ERN ReCONNET (European Reference Network on Rare and Complex Connective Tissue and Musculoskeletal Diseases) revealed that 68% of the 1093 affected people with connective tissue disease did not receive a qualified and disease-specific patient education (68.2% in Germany). However, the majority of these individuals (74%, n = 808) were interested in participating^11^. To address the specific unmet educational needs of patients with rare autoimmune diseases, different approaches have been developed: In 2004, Brown et al. reported their experience with a multimodal in-person educational program for SSc, which spanned a total of twelve hours over four weeks. The impact of the program was measured qualitatively in semi-structured interviews. Patients reported increases in knowledge as well as an increase in negative emotions. Behavioral changes could not be detected in the cohort. In 2011, Kwakkenbos and colleagues evaluated a SSc specific multimodal in-person program with a psycho-educative focus consisting of 13 individual modules over three weekends (20 h in total). 41 patients were analyzed in a pre-post design, which revealed less helplessness after the intervention and a higher acceptance of their limitations. Changes in disease knowledge were not evaluated. Poole et al. tested two similar approaches in 2013 and 2014 sending informational material (workbook and exercise DVD) via mail and offering access to online teaching units, respectively. Both interventions focused on self-management techniques, enrolling 49 patients (2013) and 16 patients (2014). In the 2013 intervention a statistically significant change in self-efficacy for pain was noted, while in the 2014 intervention significant improvements in the ability to manage care, health efficacy as well as a decrease in fatigue and depression was shown. These studies did not evaluate knowledge gains^12,13^. Lastly, in 2022 Kwakkenbos et al. developed a self-directed online self-management program to promote self-responsibility for disease management in SSc known as SPIN-SELF program. They evaluated the user-friendliness and its acceptance by the study participants. Participants were encouraged to learn online about 9 topics. The user logs showed that the program usage was low: only 2 out of 9 users logged into the program once, and 4 participants accessed none or only one of the 9 available modules of the program^14^. Several observations can be derived from these studies: Firstly, while focus groups on the conception of these interventions regularly stated a high level of informational need, an increase in disease-specific knowledge was seldom evaluated^12,13,15^. Secondly, simply providing information material is not sufficient to get participants involved with the topic^14,16^, while in comprehensive in-person programs, time investment and travel distance are reasons for non-participation for many potential participants^15,17,18^. Lastly, observational study designs were used, presumably due to small cohort sizes, and randomized controlled studies evaluating patient education in SSc are lacking.

Therefore, we conceived a randomized controlled trial investigating a two-day structured online educational seminar as a middle ground, offering engaging and specific knowledge transfer on topics relevant to SSc patients, while being accessible. The aim of this study was to gain insight into the effectiveness of the knowledge transfer, as measured by a sustained increase in knowledge through consecutive multiple-choice tests.

## Methods

We conducted a randomized controlled intervention study with a waiting list control group. The primary objective was the sustained change in the disease-specific knowledge measured by the score difference in a multiple-choice (MC) test between baseline and 3 months after intervention. The control group received a second MC test 3 months after baseline without an intervention.

### Terminology

It should be noted that the term “patient education” is defined by EULAR as "a planned interactive process to support and enable people to manage their life with an inflammatory rheumatic joint disease and optimize their health and well-being" and includes individual and/or group sessions offered face-to-face or online, which can be supplemented by telephone calls, written or multimedia materials^6,19^ This definition differs from the one commonly used in Germany, where the term ‘patient education’ does not encompass individual trainings. Furthermore, the EULAR recommends patient educational programs to be underpinned with a theoretical framework such as cognitive behavioral therapy. We opted to establish a strictly educational program. To circumvent any conflict with the specific EULAR recommendations for patient education programs, we will henceforth refer to our program as an ‘educational seminar’.

### Involvement of research partners

Two research partners were involved in the development of the study from the beginning. Both were members of the patient self-help organization Deutsche Rheuma-Liga (DRL) and trained research partners. Taking account of the EULAR recommendations, the patient organization has developed an interactive training course in which patients are prepared for their tasks and trained to become so-called "research partners"^6,19,20^. They actively participated in the design of the information texts for participants in the research project and the online questionnaires, supported the dissemination of the study call, and acted as test subjects for the online questionnaires. Additionally, they participated in the development of the educational seminar program. All accompanying documents, including presentation slides and video transcripts (patient invitation, questionnaires etc.), were reviewed for patient comprehensibility.

### Conception and content of the educational seminar

The intervention was an online educational seminar which ran over 2 days, on a Friday evening (2 h) and a Saturday morning (4 h). All participants took part in the same educational seminar at the same time. Participation was only possible with a dial-in link to the web conferencing system BigBlueButton (version 2.4, BigBlueButton Inc., Ottawa, Ontario K1S 5B6, Canada, https://bigbluebutton.org/) used for the seminar, including its chatroom function.

The seminar was composed of nine modules. On the first day, the topics ‘Pathogenesis’, ‘Disease Pattern and Organ Manifestations of SSc’ and ‘Medications and Drug Side Effects’ were discussed. The first module lasted 10 min, and the last two modules 45 min each. After the second part, a 15 min break was given. On the second day, lectures were held on the topics ‘Sport and Exercise, Stress management’, ‘Nutrition and Smoking’, ‘Osteoporosis prevention’, ‘Vaccinations and Avoidance of infections’‚ ‘Sexuality, Pregnancy and Family planning’ and ‘Self-Help, Work and Occupation’. The six modules lasted 30 min each. After the first three parts, another 30 min break was given. During active sessions and breaks attendees were allowed to use the chatroom for communication among each other and the speakers. The session concluded with a discussion and a 10-min question-and-answer period.

The lectures were developed by rheumatologists in collaboration with the patient self-help group DRL. We based our selection of topics on the EULAR recommendations and the patient information materials of the German Society for Rheumatology, patient self-help organizations (DRL, Scleroderma self-help) and the German Network Systemic Sclerosis register^21^. The physicians prepared the content of the presentations and formulated the lectures. The research partners checked the content for relevance and comprehensibility. All lectures were recorded in Microsoft 365 PowerPoint (Microsoft Corporation, Redmond, WA 98052–6399, USA, https://www.microsoft.com/) before and played back during the educational seminar. Videos were also integrated into the lectures to visualize diagnostic and therapeutic procedures. These included a pulmonary function test, bronchoscopy, capillary microscopy, and applications in physical therapy and occupational therapy. The videos were recorded solely for the educational seminar. The examinations were explained in a generally understandable way by a pulmonary function assistant, a pulmonologist, an angiologist and a physical therapist. The presentations were also reviewed by the research partners. After completing the study, the participants were given access to the videos as video on demand.

### Participants

Invitations were extended to patients with SSc through our tertiary outpatient clinic, eight collaborative rheumatological practices in Saxony-Anhalt, and across Germany via the websites of the patient self-help organization (DRL, Scleroderma self-help) and the German Network Systemic Sclerosis. After signaling interest in participation, patients gave informed consent and were enrolled. Participants were recruited from 27/04/2022 to 30/06/2022. The follow-up ended on 24/09/2023. Patients diagnosed with SSc, who are German-speaking and aged between 18 and 90, were considered suitable for inclusion. The exclusion criteria encompassed high disease activity or severe concurrent disease that would prevent seminar attendance, as well as severe mental or cognitive impairment that would hinder knowledge transfer. The spouses of patients were permitted to partake in the seminar. Patients with SSc were divided into two groups through randomization: the intervention group and the control group on the waiting list. Upon enrollment, participants were randomly allocated to these groups by our tertiary outpatient clinic utilizing external randomization lists. It should be noted that the study personnel and statisticians were not subjected to blinding.

### Instruments

All participants received access to the online questionnaires through personalized email links. For the online survey, we utilized the software LimeSurvey (version 3, LimeSurvey GmbH, 22453 Hamburg, Germany, https://www.limesurvey.org/).

*Disease-specific knowledge* was assessed by a MC test with 20 questions about disease progression, diagnosis, treatment, and lifestyle of SSc patients. The content of the MC questions was developed by three rheumatologists and reviewed for comprehensibility and relevance by the research partners. Each question provided a selection of five possible answers, with only one being correct. During the session, the participants had the opportunity to obtain all the information relevant to answering all questions correctly. A minimum of 0 and a maximum of 20 points could be scored in the test. The MC test can be found in the Supplemental Material.

The intervention group completed the MC testimmediately before and after the seminar, as well as 3 months later. The control group answered the MC test four times: 3 months before, immediately before and after the seminar, and 3 months later. The primary outcome measure was the difference in points scored before and 3 months after the seminar (intervention group) versus the same time span without an intervention (control group). The control group was created to measure or exclude any non-specific learning effects conditioned by the recurrent completion of the identical MC test. For both groups, the content of the questions always remained identical. However, the sequence of the questions and the array of the potential answers were shuffled in each repetition to additionally attenuate non-specific learning effects. There was no feedback to the participants about the correct answers or the results between each MC test.

*Demographics and baseline disease information*, including age, gender, type of SSc, duration of SSc, disease manifestation, drug therapy, comorbidities, education level and professional status, health behavior and membership of a self-help group were collected at baseline and at the end of follow-up. For items believed to be predictors of disease knowledge a multiple regression analysis was performed.

*Program evaluation questions* were asked to determine the participants’ experiences with and opinions about the educational seminar. At the end of the educational seminar, study participants were requested to evaluate the relevance of the topics and acceptance of the education format. For each item, patients had to give their opinion on a five-point Likert scale. A copy of the questionnaire (in German) is available from the authors on request.

### Power calculation and statistical analysis

#### Primary outcome and power calculation

We assumed that the mean score of the correct answers in the MC test before the intervention would be 10 and that the intervention would cause an increase to 15. The standard deviation was expected to be 2.5 in the intervention group. We calculated a group size of 30 participants for each group, with a power of 80% and a significance level of 5%.

Participants with incomplete data were excluded from the analysis. A Student’s t-test was employed to compare knowledge changes between the intervention and control group at a 5% significance level. For sensitivity analysis, we compared data from both the intervention group and the intervention part of the waiting list control group, as well as pooled data from both groups.

#### Multivariable factor analysis

A multiple linear regression was conducted to identify factors influencing the test score. The model simultaneously incorporated age, sex, disease duration, education level, and membership in a self-help group as independent variables. All p-values were interpreted in an exploratory manner.

#### Patient characteristics

Differences between groups were assessed using the student’s t-test for normally distributed variables. Wilcoxon’s test was used to compare variables before and after the intervention in the same group. The chi-square test was used to compare categorical variables of the two groups. The IBM-software SPSS Statistics (version 25, IBM Corp., Armonk, NY, USA, https://www.ibm.com/) was used for all the statistical analyses.

#### Quality criteria for MC test questions

The quality criteria for MC questions were calculated using statistical indicators such as the difficulty index (DI1) and the discrimination index (DI2). Our analysis utilized the classification of difficulty level for different ranges of the difficulty index (DI1 < 0.3 too hard, DI1 0.3 < DI1 < 0.8 moderate, DI1 ≥ 0.8 too easy) ^22^. Exam questions with a discrimination index of 0 are solved equally successfully by well-informed and less well-informed candidates. For our interpretation, we used the classification of discrimination level for different ranges of the discrimination index as proposed elsewhere (DI2 > 0.4 good, 0.2 < DI2 < 0.4 moderate, 0 < DI2 < 0.2 low, DI2 < 0 negative)^23^. The Supplemental Material contains details on the calculation of these indices.

### Research ethics

The study received approval from the ethics committee of the medical faculty of the Martin-Luther- University Halle-Wittenberg (protocol code 2021–120; date of approval 26/11/2021). The study was registered in the German Clinical Trials Register (DRKS) under the protocol code DRKS00024915 on 04/01/2022 and was conducted in accordance with the guidelines of the Declaration of Helsinki. All participants provided written informed consent. We used the CONSORT reporting guidelines^24^.

## Results

**Figure 1:**
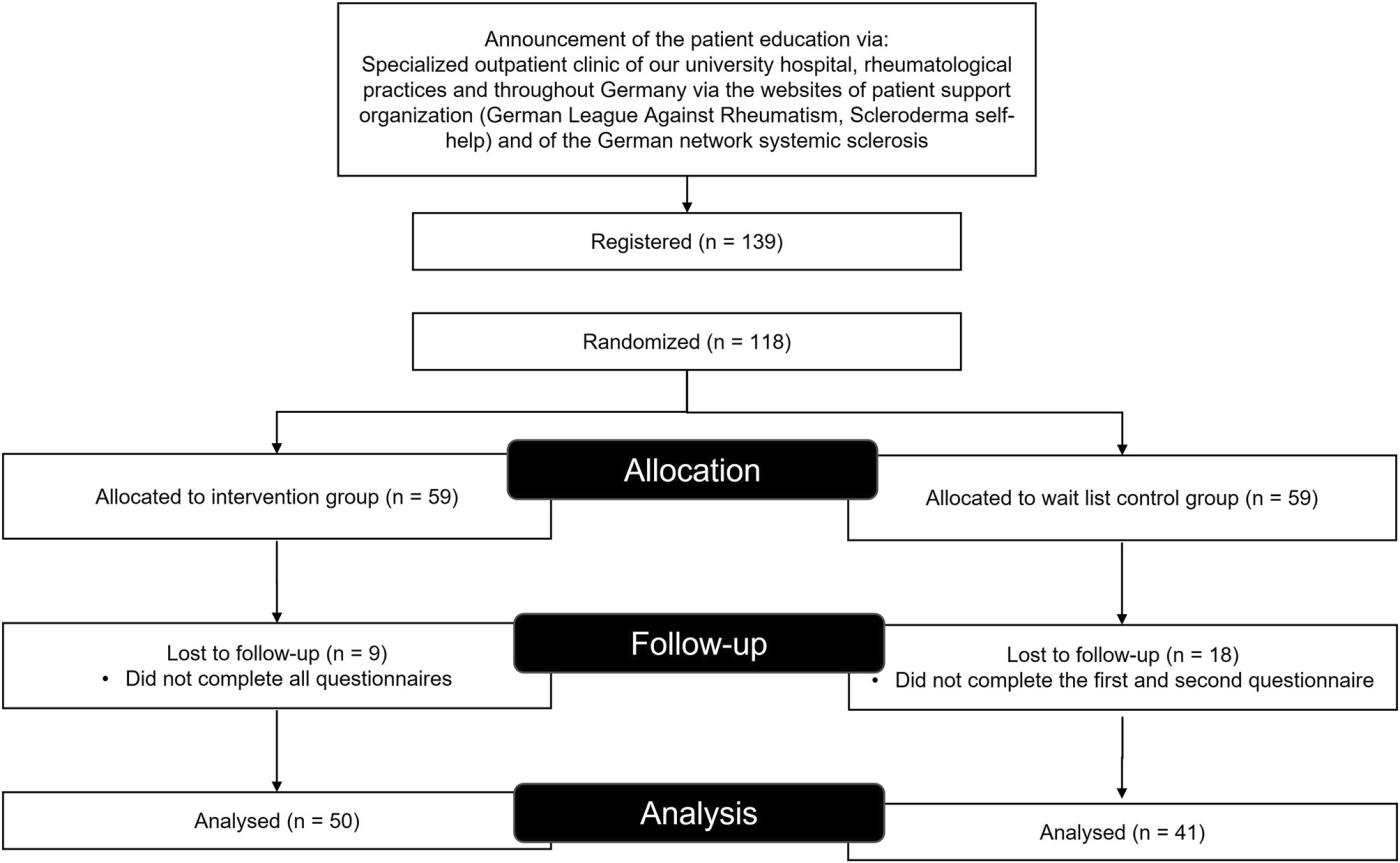
Study flowchart. 118 patients with systemic sclerosis (SSc) were randomized. Of these, in the intervention group 50 patients completed all questionnaires, in the waiting list control group 41 patients completed the first and second questionnaire for further analysis.

In total, 118 patients diagnosed with SSc were subjected to randomization, out of which 102 participated in the online educational seminar. From these participants, 91 patients successfully completed the pertinent questionnaires and were subsequently included for further analysis (refer to Fig. 1). Consequently, 50 patients were assigned to the intervention group and 41 to the control group, which was on a waiting list. The intervention group was comprised of 44 female and six male patients, while the control group included 37 female and four male patients. The average age of the participants was 55.3 years, with the intervention group averaging at 54.9 years and the control group at 55.9 years. For additional details, please refer to Supplementary Table 1.

### MC results of the participants

**Figure 2:**
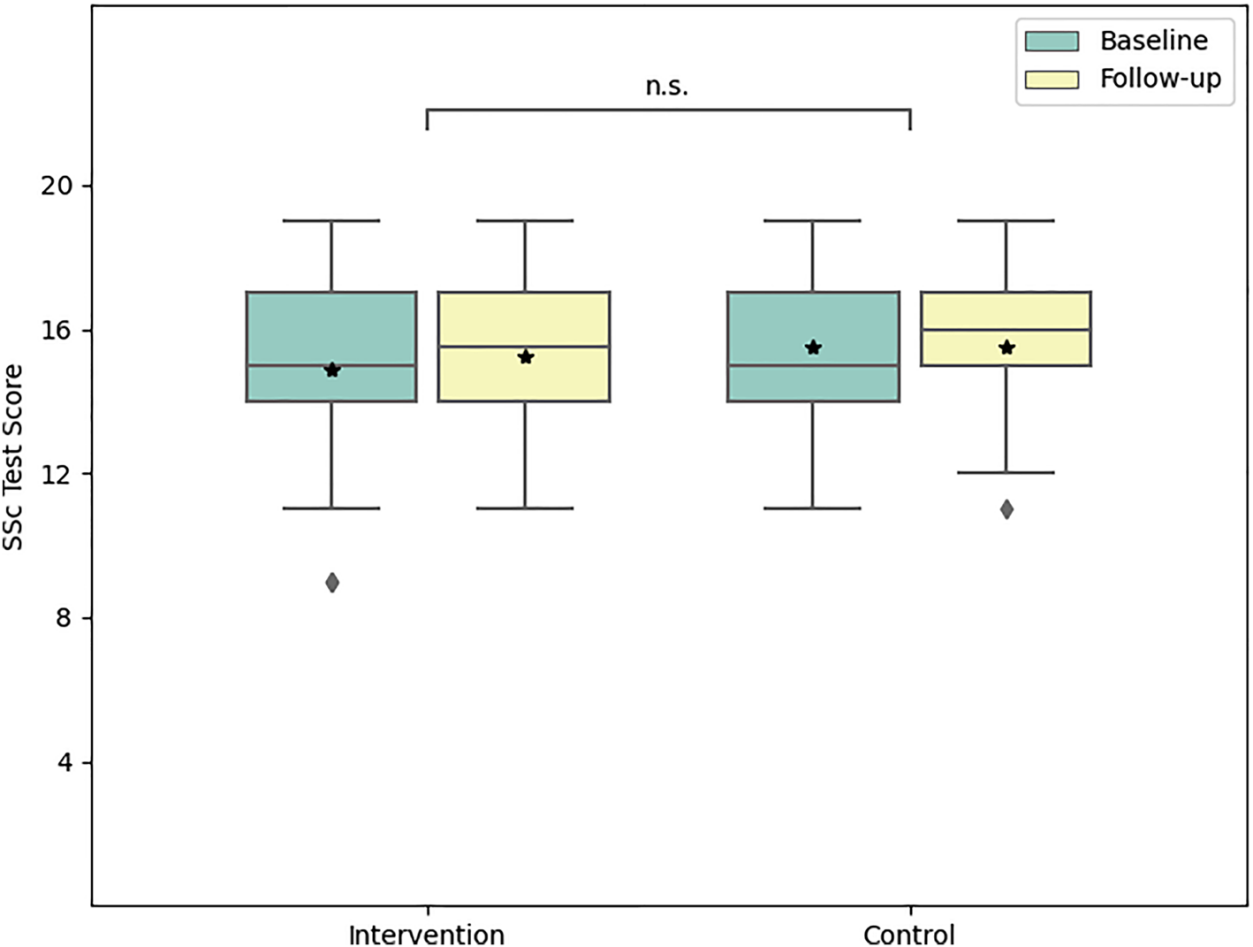
Boxplots of intervention and control group at baseline and follow-up. Group means are indicated with an asterisk; group outliers are indicated with a rhombus. The knowledge gains in the intervention group were unmodified over a three-month period despite educational seminar, test scores in the control group remained unchanged. The mean score difference between groups at follow-up was 0.34. The mean score difference between groups at follow-up was 0.34. A minimum of 0 and a maximum of 20 points could be scored in the test. *SSc* systemic sclerosis.

Prior to the intervention, the intervention group’s mean score, representing the number of correct responses, was 14.9. This score experienced an increase to 15.7 (a score difference of 0.8) subsequent to the intervention, and further adjusted to 15.2 (a difference of 0.3) three months post-intervention. The mean score difference ± standard deviation (sd) before and three months after the intervention was calculated as 0.34 ± 1.3. In the control group, the repeated answering of the questions after a three-month period without intervention did not result in an increased score on the MC test (mean score difference 0.0 ± 1.31). As the primary objective of our study, we found that the intervention, in comparison to the control group, enhanced the participants’ knowledge (mean score difference 0.34 ± 1.31, 95% confidence interval (CI) (− 0.23; 0.86), p = 0.26) (Fig. 2). For additional details, please refer to Table 1.

**Table 1:** Results of the multiple-choice test.

	Total*(n = 91)		Intervention (n = 50)		Control (n = 41)	
	M ± SD	95% CI	M ± SD	95% CI	M ± SD	95% CI
Baseline	15.2 ± 2.2	14.7, 15.7	14.9 ± 2.3	14.3, 15.5	15.5 ± 1.9	14.9, 16.1
3 month follow-up†					15.5 ± 2.0	14.9, 16.1
Post-intervention	15.9 ± 2.0	15.5, 16.3	15.7 ± 2.0	15.1, 16.3	16.1 ± 1.9	15.5, 16.7
3 month follow-up	15.4 ± 2.2	14.9, 15.9	15.2 ± 2.3	14.6, 15.8	15.7 ± 2.1	15.1, 16.3

*Second questionnaire only for the waiting list control group directly before the intervention. The mean score difference between groups at follow-up was 0.34. A minimum of 0 and a maximum of 20 points could be scored in the test.

†All participants received the intervention. The pooled data show the results of the multiple-choice test from all participants immediately before the intervention, directly afterwards, and 3 months later. M, mean; SD, standard deviation; CI, confidence interval.

#### Sensitivity analysis

**Figure 3:**
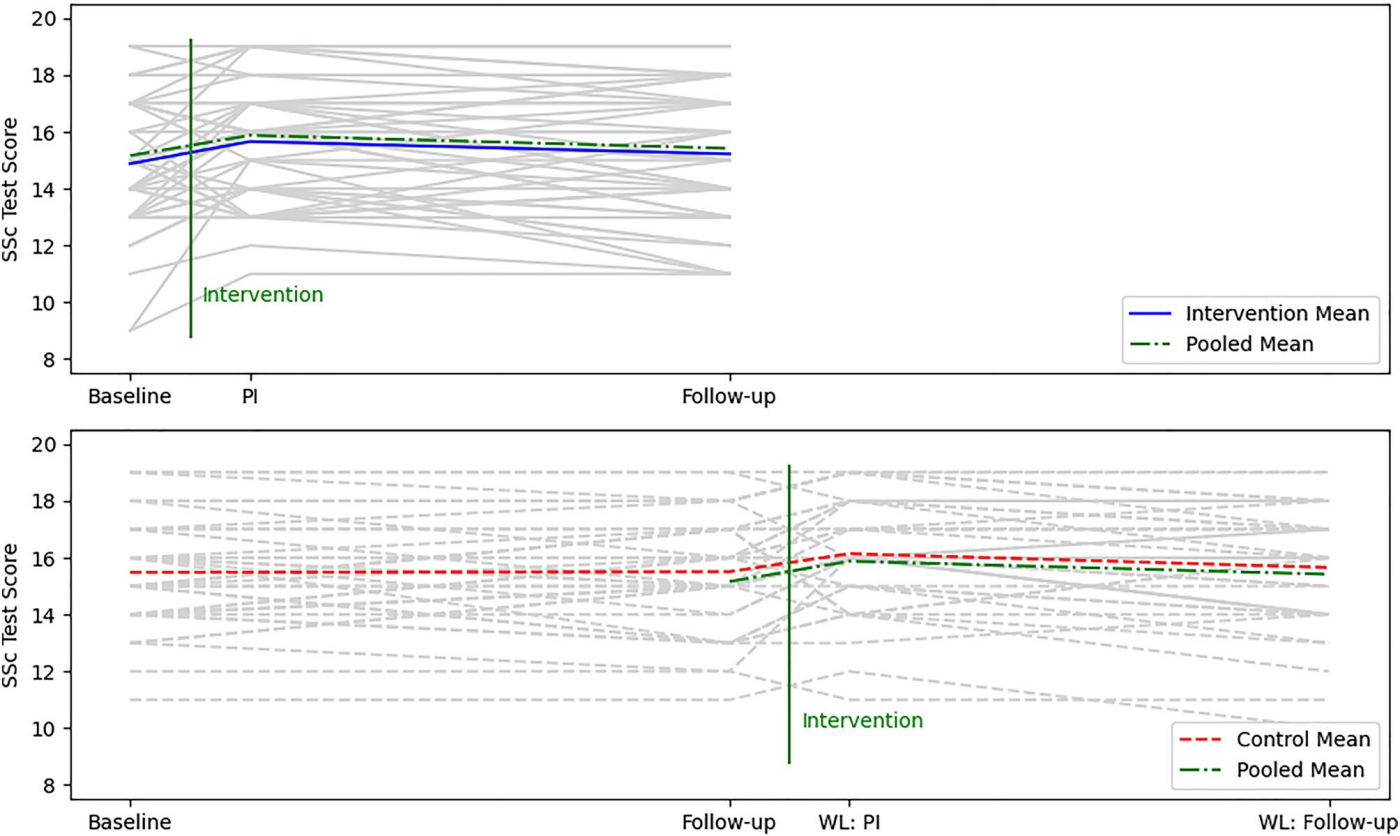
Test scores of **A** intervention group and **B** waiting list control group. The colored lines indicate the mean (dashed red line, control; solid blue line, intervention; dashed-dotted green line, pooled data). In the control group the repeated completion of the questions without intermediate intervention did not lead to an increase in correct answers from baseline to follow-up. The mean score difference between groups at follow-up was 0.34. A minimum of 0 and a maximum of 20 points could be scored in the test. *PI* post-intervention, *WL* PI waiting list control group post-intervention, *WL* follow-up, waiting list control follow-up, *SSc* systemic sclerosis.

Before the intervention, the mean score did not differ between the intervention and waiting list control group. The intervention improved the test results of the control group equally to those of the intervention group. Before the intervention, the mean score in the pooled data was 15.2 ± 2.2. Immediately after the intervention, the score increased to a mean of 15.9 ± 2.0. Three months later, the value declined with a mean of 15.4 ± 2.2 correct answers (Fig. 3).

### Multivariable factor analysis

To identify factors influencing test scores, a multiple linear regression was performed. The analysis revealed that the duration of illness (in years) (β = 0.05, 95% CI (0.01; 0.09), p = 0.02), membership in a self-help group (β = 1.12, 95% CI (0.12; 2.12), p = 0.03), and level of education (general higher education entrance qualification vs. less than high school β = 1.49; 95% CI (0.38; 2.59), p = 0.01; completed higher education vs. less than high school β = 1.11, 95% CI (0.1; 2.12), p = 0.03) are positive predictors of a higher score before patient education. Age (in years) was a negative influencing factor (β =—0.06, 95% CI (− 0.11;—0.02), p = 0.00). Gender had no influence on the score (female β = 0.08, 95% CI (− 1.23; 1.39), p = 0.90) (Table 2).

**Table 2:** Results of the linear regression.

	Regression coefficient	Lower 95% CI	Upper 95% CI	P value
(Constant)	16.536	13.754	19.318	< .001
Sex				
Male (reference)				
Female	.081	− 1.232	1.393	.903
Duration of SSc	.047	.008	.086	.018
Education				
Less than high school (reference)				
High school	1.486	.384	2.587	.009
University degree	1.108	.099	2.118	.032
SSc self-help group membership, Yes	1.123	.120	2.126	.029
Age	− .064	− .106	− .021	.004

*SSc* systemic sclerosis, *CI* confidence interval.

Overall, the model accounted for only a small proportion of the variance in the baseline knowledge test score (corrected r^2^ = 0.17), indicating that the variables considered did not effectively predict baseline patient knowledge. Membership in a self-help group was the most important influencing factor. In our dataset, 70 (76.9%) out of 91 participants who were already members of a self-help group had a mean score of 15.4 points before the seminar. The remaining 21 participants had a mean score of 14.2 points. The two lowest scoring participants (9 points) before the seminar were also not members of a self-help group.

### Difficulty and discrimination index of the MC questions

There were altogether 20 MC questions. The results shown in Supplementary Table 2 offers five out of 20 questions attained a moderate discrimination index, namely Q9, Q17, Q18, Q19 and Q20. These values indicate that the questions are acceptable and similar questions can be used in future examinations. On the other hand, Q1, Q2, Q5, Q6, Q7, Q10, Q12, Q15 and Q16 obtained a low discrimination index which implicates that these questions were not able to differentiate the groups of participants with more and less knowledge. At least there were six questions (Q3, Q4, Q8, Q11, Q13, Q14) without discrimination, because all participants gave the correct answer. The difficulty index for the questions, in descending order, are Q1-Q4, Q6, Q8, Q10-Q17 with a difficulty index > 0.8, followed by Q5, Q7, Q9, Q18-20 with a value between 0.3 < DI1 < 0.8 (Supplementary Table 2). A higher difficulty index indicates an easier question, while a lower difficulty index suggests a more challenging question. In our test, we found that most of the participants had difficulty in answering question Q9 with only 34.1% ticking the correct answer. This was followed by Q19 with an achievement of 50.6%. Next was the Q20 with an achievement 57.1%. The easiest questions achieved a percentage of correct answers up to 100%.

### Analysis of the program evaluation

Apart from the MC test, our survey contained questions addressing the evaluation of the online educational seminar. The majority of participants (85%) found the seminar helpful, and 92% would recommend it to others. After the two days 85% felt better informed than before. 90% would attend such a seminar again. The online format was well received by the participants, 95% found it suitable and the implementation of the training very good. The most common reasons for preferring an online format were saving time and money, family suitability and the possibility to participate from anywhere. About two-thirds of participants (60%) prefer the online seminar format and 98% would like to attend an online patient education again (Supplementary Fig. 1).

## Discussion

### Study design and recruitment strategy

We opted for a waiting list control group design to rule out a non-specific knowledge gain through repeated testing. This meant, however, that participants were not blinded to their allocated group. Those who received two questionnaires before the intervention knew that they were in the waiting list control group. However, this fact did not motivate the participants to train themselves in the months before the intervention. There was no increase in knowledge in the waiting list control group before the intervention. The point dynamics in the MC test after the intervention did not differ in both groups, so that one cannot assume an overestimation of treatment effects in the waiting list control group.

Concerning the recruitment strategy, we assume two sources of possible selection bias: Firstly, announcements of the educational seminar were – among other channels – spread via self-help groups. Thus, there is a chance we disproportionately reached patients who are already well connected and actively managing their disease. Secondly, the proportion of women among the participants was high at 89% and does not correspond to the gender distribution of the disease. Our experience shows that women are more open to participate in training and more likely to share their experiences with the disease.

### Disease-specific knowledge gain

An improvement in the test score after the intervention was believed to be a disease-specific knowledge gain. The educational seminar resulted in a small but not significant sustained increase in knowledge. We identified two possible reasons for this: Firstly, the choice of MC questions could have resulted in existing knowledge gains being insufficiently measurable. Patients who were already well-informed did not significantly improve their knowledge. However, a portion of participants who started with a lower score were able to improve considerably (see Fig. 3). Therefore, we speculate that participants who were already well-informed at baseline may not have been able to demonstrate their knowledge gains in the MC test after the intervention. This ceiling effect occurs when the items in the test are not evenly distributed across the difficulty range: 15 out of 20 questions (75%) had a DI1 above 0.8 and were therefore regarded as too easy. Furthermore, analysis of the questions revealed that 13 questions did not provide sufficient discriminatory power (DI2 < 0.2) between well-informed and less-informed participants. Here too, the ceiling effect might play a role. The questions were previously checked for comprehensibility by the research partners. We refrained from a preliminary test run of the questions on SSc patients, as this would have considerably reduced the number of eligible participants for the actual seminar. On a sidenote, online tests carry the risk that participants search for answers to the MC questions on the internet. However, we have no means to test for this.

Secondly, the seminar itself by its online format, chosen contents and scope might have hampered knowledge transfer. We identified several improvable factors in retrospect: During the lectures, lively discussions emerged in the chat function. Participants were constantly asking questions to the speakers and exchanging information among themselves. Here, attention was certainly diverted from the lectures. Prevalent distractions may also be indicated by the fact that after the seminar, the moderately difficult questions continued to be answered incorrectly even by the well pre-informed participants. Furthermore, on the second day of the seminar lectures ran for 4 h in total with a 30-min break. In the program evaluation, participants indicated that this part was too demanding to follow. As a consequence of both observations, we conclude that knowledge transfer might have been hampered by offering distractions in form of a chat function and too long blocks of seminar content. Implementing more and longer breaks might address both issues: the need for personal communication with experts and information exchange, as well as the necessary rest to actively follow the lectures. General considerations on the seminar concept and online format are found below.

In summary, we believe that the primary factor for not achieving the main endpoint of the study was the MC test, which was too easy and thus suffered from a ceiling effect. However, there is room for improvement in the seminar itself.

### Predictors of disease knowledge

With a mean MC test score at baseline of 15.2 points, participants were already well informed about their disease before the seminar. In our study, membership in a SSc self-help group and a higher level of education emerged as the most significant independent positive predictors for test scores, with a β of 1.1 and ~ 1.3 respectively. Age was a negative predictor, with approximately − 1.3 points per 20 years of age difference.

While the positive influence of a higher education level is unsurprising and has been shown in other investigations^25,26^, the relevant positive influence of a self-help group is worth exploring: In our cohort, 76.9% were members of a SSc self-help group, which could partly be attributed to our recruitment strategy. Nevertheless, scleroderma patients seem to be well connected in Germany. Karp et al. found in their literature review that predominantly white, female patients with an average age of more than 50 years participate in peer support^27^. This is also consistent with our data. Studies conducted in Canada, the United States, and Europe have examined the reasons for participation in SSc self-help groups. The responses were similar: sharing information on treatment, symptom management and social support^27^. Participants in patient education programs report on improved knowledge about their disease and they appreciate the opportunity to share their experiences with other affected individuals and physicians^11^. Haythornthwaite and colleagues reported that one-half of 142 patients with scleroderma had a mild depression^28^, consistent with earlier finding from Roca et al.^29^. Involvement in a social network like a self-help group may protect against depressive symptoms and improve physical and psychosocial adaptation in scleroderma^29^.

Self-help groups have established structures to facilitate knowledge transfer. For instance, in the DRL, train-the-trainer programs are offered to physicians and healthcare professionals to impart evidence-based concepts for needs-based patient education. At the peer level, Thombs et al. developed a support group leader education program to enhance the knowledge disseminated in self-help groups. The aim of the study was to improve the quality of education and to reduce harms from dissemination of inaccurate information. After a 13-week course with 13 60–90 min sessions, the group leader’s self-efficacy scores were substantially higher. They did not evaluate whether participation in the training program led to improvements in support group quality^30^. Still, a formalized approach to knowledge transfer among peers seems advantageous.

Disease specific knowledge gains through self-help groups have been shown for other chronic conditions: McCarron et al. studied the effects of a support group for patients with rheumatoid arthritis. For that purpose, sessions of six-monthly self-help group meetings lasting approximately 60 min, with the researcher as a participant observer, were analyzed. They ascertained an empowerment of the participants with increased knowledge and self-efficacy^31^. A study involving breast cancer patients revealed that membership in a self-help group enhances disease-related knowledge^32^. These findings validate our results.

### Online versus in-person programs

We opted to conduct the educational seminar online to reach a larger audience of SSc patients. With a local in-person seminar we would not have been able to recruit a sufficient sample size as indicated by the power calculation. However, we were aware of potential disadvantages of the online delivery mode: Firstly, due to the online format few patients with little or no access to the internet may not have been able to participate. In contrast, the use of an online format makes it possible for people who cannot participate in an in-person program for example due to forbidding travel costs as in-person programs are usually only offered by tertiary centers.

Secondly, beyond distraction offered through the chat function as discussed above, the possibility of participating in a training course from home entails the risk of being distracted by everyday matters. A larger knowledge gain was demonstrated in educational seminars of the same length for granulomatosis with polyangiitis and systemic lupus erythematosus patients taught in-person in 2019^33^. In another study, 102 vasculitis patients were trained by experienced trainers in closed groups of 10–15 participants. Group discussions with interaction and exchange of own experience alternated with short lectures. Before the intervention, the participants scored 20.5 and four weeks after the intervention, they obtained 29 out of 45 points (p < 0.05)^34^. Warsi and colleagues describe in their systematic review for self-management education programs in chronic disease that interventions that incorporated face-to-face education were more effective than video programming, telephone contacts, audiocassettes, and written materials. The duration of the training program, the number of lessons and the format were not associated with improved effectiveness^35^.On the other hand, Friedman et al. found that the use of computer technology could be a potent teaching strategy with positive effects on patient knowledge. They suggest that different teaching strategies (computer, audiotapes, videotapes, written materials, demonstrations) should be used in combination and were similarly successful^36^.

After all, it seems there is an inherent tradeoff between reach and effect size with online formats being accessible to more patients with rare rheumatic diseases and local in-person formats having a higher effect in terms of sustained knowledge transfer.

### Strengths and limitations

The study—and the conception of the seminar in particular—emphasized on participative research. We report on the largest SSc cohort in the context of a patient education intervention and were able to utilize a randomized controlled design. The adherence to the program among intervention participants was high, with complete data from 77% of participants. The educational seminar addresses an established need for patients with rare autoimmune disease and strikes a good balance between resource intensive and lengthy in-person programs and unguided access to informational material. Participants evaluated the educational seminar as excellent.

Although a small sustained knowledge transfer was demonstrated this effect was not statistically significant and thus the primary endpoint was not met. Two main limitations arguably contributed to missing the objective: As indicated by distractive and thereby counterproductive ample chatting during the lectures, we underestimated (and maybe undervalued) the participants need for communication. Consequently, we did not allocate enough time to breaks and discussion sessions to satisfy this need outside of lectures. Furthermore, the MC test was too easy. As a result, it suffered from a ceiling effect and already well-informed participants were unable to demonstrate their knowledge gains.

Despite the excellent evaluation from participants, we were unable to demonstrate statistically significant knowledge gains for SSc patients through the online educational seminar. The study conclusively showed that membership in a self-help group is an independent positive predictor of disease knowledge. In practice, a tradeoff between effectiveness and accessibility must be accepted when conducting online educational programs.

### Supplementary Information

Supplementary Information.

## Data Availability

The dataset and analysis documents are available from the corresponding author upon reasonable request.
