# Supplementary material for: An online, two-day educational seminar had no impact on disease-specific knowledge in patients with systemic sclerosis
Source: Sci Rep. 2024 Jun 14;14:13767. doi: 10.1038/s41598-024-64532-4 (PMC11178768; doi:10.1038/s41598-024-64532-4)
Supplement: Supplementary file 1 — Supplementary Information. [file 41598_2024_64532_MOESM1_ESM.docx]

# Supplemental material:

***Supplementary table 1.*** *Baseline characteristics of participants.*

| Characteristics | Total (n = 91) | Intervention (n = 50) | Control (n = 41) |
| --- | --- | --- | --- |
| Age, y (M ± SD) | 55.3 ± 11.2 | 54.9 ± 10.3 | 55.9 ± 12.3 |
| Sex, n (%)  Men  Women | 10 (11.0)  81 (89.0) | 6 (12.0)  44 (88.0) | 4 (9.8)  37 (90.2) |
| BMI (kg/m²), (M ± SD) | 24.6 ± 5.3 | 24.4 ± 4.8 | 24.9 ± 5.8 |
| Education, n (%)  Less than high school  High school  University degree | 25 (27.5)  25 (27.5)  36 (39.6) | 15 (30.0)  14 (28.0)  18 (36.0) | 10 (24.4)  11 (26.8)  18 (43.9) |
| Employment, n (%)  Employed/self-employed  Retired | 53 (58.2)  25 (27.5) | 35 (70.0)  10 (20.0) | 18 (43.9)  15 (36.6) |
| Smoker, n (%)  Current smoker  Non-current smoker  Past smoker | 3 (3.3)  35 (38.5)  31 (34.1) | 2 (4.0)  18 (36.0)  16 (32.0) | 1 (2.4)  17 (41.5)  15 (36.6) |
| SSc self-help group membership, n (%) | 70 (76.9) | 40 (80.0) | 30 (73.2) |
| Duration of SSc, y (M ± SD)  Age at disease onset, y (M ± SD) | 9.7 ± 12.1  45.6 ± 13.2 | 9.8 ± 12.5  45.1 ± 12.1 | 9.5 ± 11.7  46.3 ± 14.6 |
| SSc subtype, n (%)  Limited cutaneous SSc  Diffuse cutaneous SSc  SSc sine scleroderma  Circumscribed scleroderma  Overlap-Syndrome | 36 (39.6)  20 (22.0)  7 (7.7)  1 (1.1)  14 (15.4.) | 20 (40.0)  10 (20.0)  6 (12.0)  0  7 (14.0) | 16 (39.0)  10 (24.4)  1 (2.4)  1 (2.4)  7 (17.1) |
| SSc manifestation, n (%)  Skin involvement  Hands  Forearm ± upper arm  Trunk  Face  Digital ulcers, teleangiectasias  Lung involvement  Kidney involvement  Cardiac involvement  Joints ± muscular involvement  Digestive tract involvement | 86 (94.5)  40 (44.0)  15 (16.5)  57 (62.6)  41 (45.1)  39 (42.9)  7 (7.7)  16 (17.6)  70 (76.9)  53 (58.2) | 47 (94.0)  21 (42.0)  6 (12.0)  31 (62.0)  25 (50.0)  21 (42.0)  3 (6.0)  11 (22.0)  38 (76.0)  29 (58.0) | 39 (95.1)  19 (46.3)  9 (22.0)  26 (63.4)  16 (39.0)  18 (43.9)  4 (9.8)  5 (12.2)  32 (78.0)  24 (58.5) |
| Autoantibodies, n (%)  Anti-centromere  Anti-Scl70  Anti-RNA pol III  Anti-U1RNP  Anti-PMScl | 13 (14.3)  39 (42.9)  1 (1.1)  1 (1.1)  3 (3.3) | 7 (14.0)  22 (44.0)  0  0  2 (4.0) | 6 (14.6)  17 (41.5)  1 (2.4)  1 (2.4)  1 (2.4) |
| Comorbidities, n (%)  Arterial hypertension  Hypercholesterinaemia  Osteoarthritis  Diabetes mellitus  Osteoporosis  Heart failure  Myocardial infarction  Pulmonary disease  Stroke  Malignancy  Mental illness | 19 (20.9)  27 (29.7)  37 (40.7)  3 (3.3)  20 (22.0)  14 (15.4)  3 (3.3)  25 (38.5)  3 (3.3)  6 (6.6  19 (20.9)) | 8 (16.0)  17 (34.0)  21 (42.0)  1 (2.0)  13 (26.0)  8 (16.0)  2 (4.0)  13 (26.0)  3 (6.0)  1 (2.0)  10 (20.0) | 11 (26.8)  10 (24.4)  16 (39.0)  2 (4.9)  7 (17.1)  6 (14.6)  1 (2.4)  12 (29.3)  0  5 (12.2)  9 (22.0) |
| Current drug exposure, n (%)  CYC  MTX  AZA  MMF  Glucocorticoids  Calcium channel blockers  Prostacycline  ETR antagonists or PDE inhibitors  Antifibrotic agents  Proton pump inhibitors  ACE-inhibitors | 1 (1.1)  21 (23.1)  2 (2.2)  16 (17.6)  12 (13.2)  7 (7.7)  13 (14.3)  22 (24.2)  4 (4.4)  45 (49.5)  11 (12.1) | 0  12 (24.0)  1 (2.0)  8 (16.0)  5 (10.0)  5 (10.0)  8 (16.0)  13 (26.0)  3 (6.0)  23 (46.0)  4 (8.0) | 1 (2.4)  9 (22.0)  1 (2.4)  8 (19.5)  7 (17.1)  2 (4.9)  5 (19.2)  9 (22.0)  1 (2.4)  22 (53.7)  7 (17.1) |

Data are shown as M (mean) ± SD (standard deviation) or number (%) of patients. BMI, body mass index; SSc, systemic sclerosis; CYC, cyclophosphamide; MTX, methotrexate; AZA, azathioprine; MMF, mycophenolate mofetil; ETR, endothelin receptor; PDE, phosphodiesterase; ACE, angiotensin-converting-enzyme

***Supplementary table 2.*** *Analysis and interpretation of difficulty and discrimination index for the multiple-choice test systemic sclerosis.*

| **Question No.** | **Difficulty index** | **Interpretation** | **Discrimination Index** | **Interpretation** |
| --- | --- | --- | --- | --- |
| Q1 | 0.95 | too easy | 0.14 | Low positive discrimination |
| Q2 | 0.99 | too easy | 0.05 | Low positive discrimination |
| Q3 | 1.00 | too easy | - | No discrimination |
| Q4 | 0.92 | too easy | -0.01 | No discrimination |
| Q5 | 0.76 | moderate | 0.11 | Low positive discrimination |
| Q6 | 0.97 | too easy | 0.12 | Low positive discrimination |
| Q7 | 0.77 | moderate | 0.11 | Low positive discrimination |
| Q8 | 1.00 | too easy | - | No discrimination |
| Q9 | 0.34 | moderate | 0.25 | Average positive discrimination |
| Q10 | 0.82 | too easy | 0.19 | Low positive discrimination |
| Q11 | 0.96 | too easy | -0.01 | No discrimination |
| Q12 | 0.88 | too easy | 0.19 | Low positive discrimination |
| Q13 | 1.00 | too easy | - | No discrimination |
| Q14 | 0.90 | too easy | 0.01 | No discrimination |
| Q15 | 0.96 | too easy | 0.13 | Low positive discrimination |
| Q16 | 0.92 | too easy | 0.12 | Low positive discrimination |
| Q17 | 0.85 | too easy | 0.23 | Average positive discrimination |
| Q18 | 0.79 | moderate | 0.26 | Average positive discrimination |
| Q19 | 0.51 | moderate | 0.31 | Average positive discrimination |
| Q20 | 0.57 | moderate | 0.33 | Average positive discrimination |

**
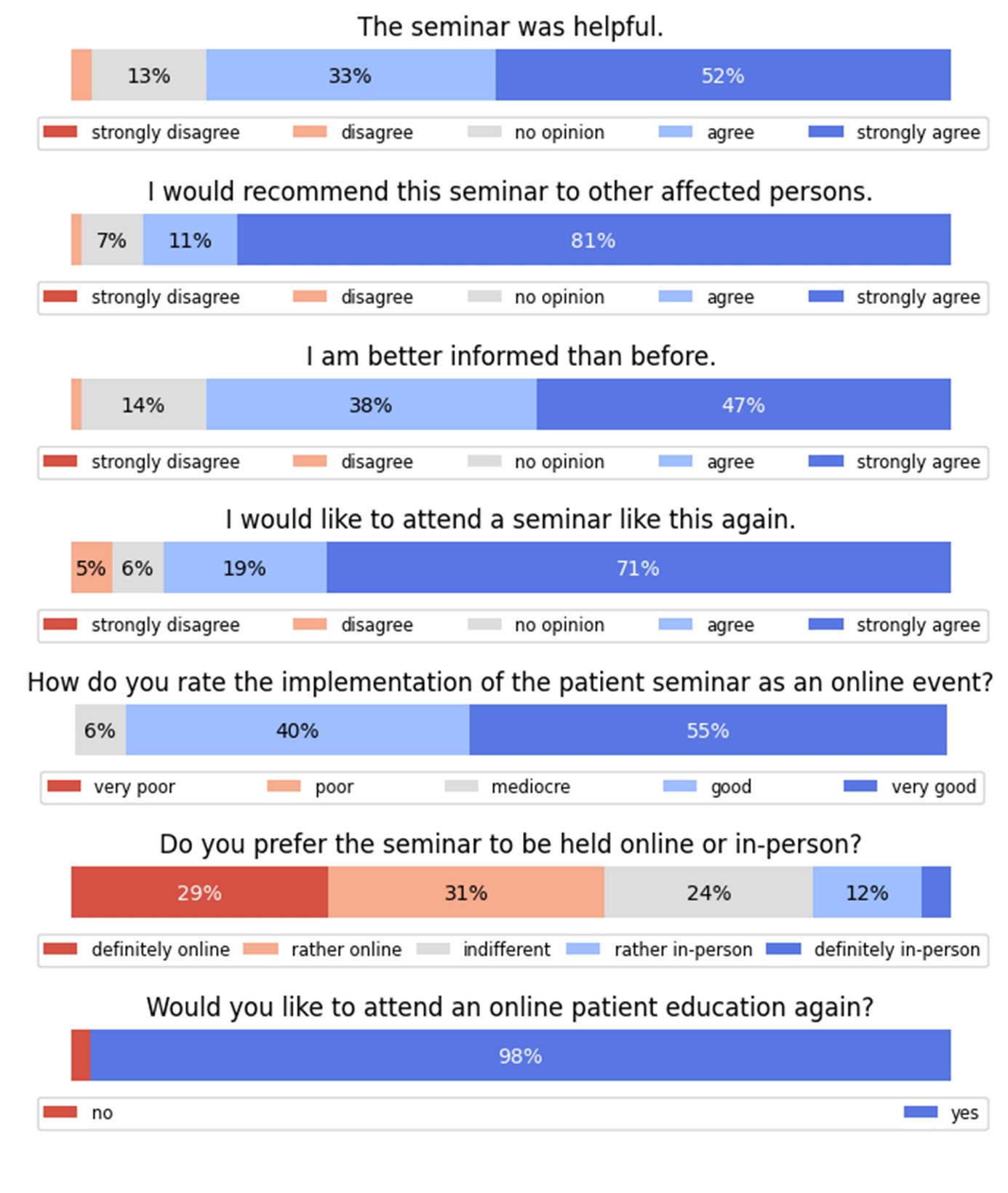
**

**Supplementary figure 1.** Evaluation and acceptance of the online educational seminar

Frequencies of responses including gradation of intensity.

**MC test**

**1. Which statement about scleroderma is correct?**

a) [ ] Scleroderma mainly affects men

b) [ ] Scleroderma can only be detected by blood tests

c) [ ] Scleroderma can affect the skin, kidneys, and lungs

d) [ ] It is a hereditary disease

e) [ ] The disease is very common in Germany

**2. In which area does scleroderma usually first manifest?**

a) [ ] On the fingers and hands

b) [ ] On the spine

c) [ ] In the gastrointestinal tract

d) [ ] In the cardiovascular system

e) [ ] On the nose and paranasal sinuses

**3. Which statement about scleroderma is correct?**

a) [ ] Scleroderma can affect the heart.

b) [ ] Scleroderma heals after about 5-10 years.

c) [ ] Scleroderma is contagious.

d) [ ] Scleroderma only occurs in Europe.

e) [ ] Scleroderma does not cause pain.

**4. Which measure for diagnosis is not useful?**

a) [ ] Nailfold microscopy / capillary microscopy

b) [ ] Bone marrow puncture

c) [ ] X-ray of the hands

d) [ ] Laboratory examination of the blood for antibodies

e) [ ] Lung function test

**5. At what age does scleroderma most commonly occur?**

a) [ ] 0 – 30 years

b) [ ] 30 – 50 years

c) [ ] 50 – 80 years

d) [ ] Over 80 years

e) [ ] Scleroderma occurs equally frequently at all ages.

**6. Which statement about the "secondary Raynaud's syndrome" is correct?**

a) [ ] It only affects the face

b) [ ] It cannot be treated with medication

c) [ ] It is mainly triggered by cold

d) [ ] It can be caused by sunlight

e) [ ] It only occurs in women

**7. Which statement about the therapy of scleroderma is correct?**

a) [ ] Antibiotics are mainly used at the beginning of therapy

b) [ ] Methotrexate is a biotechnically produced antibody

c) [ ] Cortisone (e.g., prednisolone) lowers blood sugar and blood pressure at high doses

d) [ ] Physiotherapy is not used in therapy

e) [ ] Mycophenolate mofetil can be used in case of lung involvement

**8. What can be seen in a computed tomography of the thorax ("layer X-ray" of the lung) in scleroderma?**

a) [ ] Pulmonary fibrosis (connective tissue in the lung)

b) [ ] Rib fractures

c) [ ] A fluid accumulation in the pleura

d) [ ] An air accumulation in the intercostal space

e) [ ] A severe enlargement of the heart

**9. Which statement about ACE inhibitors is correct?**

ACE inhibitors…

a) [ ] Promote the excretion of water

b) [ ] Increase blood pressure when it is severely lowered

c) [ ] Protect the kidneys

d) [ ] Can also be taken during pregnancy

e) [ ] Are primarily prescribed for the treatment of lung problems

**10. Which statement about the treatment and prevention of accompanying symptoms of scleroderma is correct?**

a) [ ] Treatment with prednisolone can lead to an increased formation of bone tissue

b) [ ] Treatment of scleroderma with prednisolone, azathioprine or methotrexate can lead to an increased occurrence of intestinal sluggishness (constipation)

c) [ ] Vaccinations against viral flu are not recommended

d) [ ] Manual lymphatic drainage is helpful for many patients with scleroderma

e) [ ] Affected persons should generally avoid sports

**11. What can worsen the disease activity of scleroderma?**

a) [ ] Prolonged UV radiation (sunlight)

b) [ ] Smoking

c) [ ] Sauna visits

d) [ ] Contraceptives (pill)

e) [ ] Low-fiber diet

**12. The increase of which component in the urine is an indication of kidney involvement in scleroderma?**

a) [ ] Oxygen

b) [ ] Proteins

c) [ ] Calcium

d) [ ] Glucose (sugar)

e) [ ] Bacteria

**13. Which statement about the causes of scleroderma is correct?**

a) [ ] Scleroderma is caused by high blood pressure and occupational stress

b) [ ] Excessive use of sunscreens and cosmetics triggers scleroderma

c) [ ] In scleroderma, there is an increased deposition of connective tissue and occlusions of small blood vessels

d) [ ] Excessive consumption of cereals (due to the protein gluten) significantly promotes the development of the disease

e) [ ] Scleroderma is caused by a deficiency of vitamins and minerals

**14. Which medication is specifically used for gastrointestinal symptoms, such as esophagitis?**

a) [ ] Glucocorticoids, like cortisone

b) [ ] Calcium antagonists, like nifedipine

c) [ ] Azathioprine

d) [ ] Endothelin receptor antagonists, like bosentan

e) [ ] Proton pump inhibitors, like omeprazole

**15. What is a sign of pulmonary hypertension?**

a) [ ] Eyelid edema

b) [ ] Shortness of breath

c) [ ] Chest pain

d) [ ] Mucous sputum

e) [ ] Pallor

**16. Where does calcinosis cutis (skin calcification) most commonly occur?**

a) [ ] In the chest area

b) [ ] In the abdominal area

c) [ ] In the genital area.

d) [ ] On the arms and hands

e) [ ] In the oral cavity

**17. Which examination should be regularly repeated in patients with scleroderma?**

a) [ ] Heart ultrasound

b) [ ] Kidney ultrasound

c) [ ] Colonoscopy

d) [ ] Stool examination

e) [ ] Skin biopsy

**18. Which organs are crucial for the course of scleroderma?**

a) [ ] Skin and joints

b) [ ] Spleen and liver

c) [ ] Brain and nervous system

d) [ ] Lungs and kidneys

e) [ ] Muscles and bones

**19. Which antibodies are typical for limited cutaneous scleroderma?**

a) [ ] anti-ds-DNA antibodies

b) [ ] anti-Scl-70 antibodies

c) [ ] anti-centromere antibodies

d) [ ] anti-CCP antibodies

e) [ ] anti-gliadin antibodies

**20. What special side effect can occur during treatment with cyclophosphamide (Endoxan®)?**

a) [ ] Increase in sperm count

b) [ ] Hearing disorders

c) [ ] Swallowing disorders

d) [ ] Taste disorders

e) [ ] Bloody cystitis

**Quality of multiple choice questions**

The item difficulty is defined as the proportion of correct answers. The difficulty index (DI1) is calculated by

$$DI1= \frac{N_{1}}{N}$$

with N_1_ being the number of correct answers and N being the total number of participants taking the test. The value varies between 0=nobody answered the question correctly and 1=all answered the question correctly. The level of difficulty depends primarily on the question and the test group. Not all participants will be able to answer difficult questions correctly. Values between 0.3 and 0.9 are aimed for. For our analysis, we used the classification of difficulty level for different ranges of the difficulty index (DI1 < 0.3 too hard, DI1 0.3 < DI1 < 0.8 moderate, DI1 ≥ 0.8 too easy) [22].

The item discrimination index (DI2) analyses how powerful a question is in distinguishing high-achieving participants from low-achieving participants. It is defined as the difference in percentages of correct answers to an item between the top quartile and the bottom quartile participants,

$$DI2=\frac{(N_{H}-N_{L})}{\frac{N}{4}}$$

with N_H_ and N_L_  being the numbers of correct answers in the top and bottom quartile and N being the total number of participants. The value ranges from -1 to 1. An exam question with a discrimination index of 0 is solved equally successfully by well-informed and less well-informed candidates. Questions with a positive discrimination index are answered better by well-informed candidates than by less well-informed ones. The discrimination index of a question should be positive and preferably 0.3 or more. For our interpretation, we used the classification of discrimination level for different ranges of the discrimination index as proposed elsewhere (DI2 > 0.4 good, 0.2 < DI2 < 0.4 moderate, 0 < DI2 < 0.2 low, DI2 < 0 negative) [23].
